# Application of the Variance Delay Fuzzy Approximate Entropy for Autonomic Nervous System Fluctuation Analysis in Obstructive Sleep Apnea Patients

**DOI:** 10.3390/e22090915

**Published:** 2020-08-21

**Authors:** Yifan Li, Shan Wu, Quanan Yang, Guanzheng Liu, Leijiao Ge

**Affiliations:** 1 Department of Biomedical Engineering, School of Biomedical Engineering, Sun Yat-sen University, Guangzhou 510275, China; liyf56@mail2.sysu.edu.cn (Y.L.); wush69@mail2.sysu.edu.cn (S.W.); yangqan@mail2.sysu.edu.cn (Q.Y.); liugzh3@163.com (G.L.); 2 School of Electrical and Information Engineering, Tianjin University, Tianjin 300072, China

**Keywords:** obstructive sleep apnea (OSA), autonomic nerve system (ANS) fluctuation, OSA severity analysis, heart rate variability (HRV), variance delay fuzzy approximate entropy (VD_fApEn)

## Abstract

Obstructive sleep apnea (OSA) is a fatal respiratory disease occurring in sleep. OSA can induce declined heart rate variability (HRV) and was reported to have autonomic nerve system (ANS) dysfunction. Variance delay fuzzy approximate entropy (VD_fApEn) was proposed as a nonlinear index to study the fluctuation change of ANS in OSA patients. Sixty electrocardiogram (ECG) recordings of the PhysioNet database (20 normal, 14 mild-moderate OSA, and 26 severe OSA) were intercepted for 6 h and divided into 5-min segments. HRV analysis were adopted in traditional frequency domain, and nonlinear HRV indices were also calculated. Among these indices, VD_fApEn could significantly differentiate among the three groups (*p* < 0.05) compared with the ratio of low frequency power and high frequency power (LF/HF ratio) and fuzzy approximate entropy (fApEn). Moreover, the VD_fApEn (90%) reached a higher OSA screening accuracy compared with LF/HF ratio (80%) and fApEn (78.3%). Therefore, VD_fApEn provides a potential clinical method for ANS fluctuation analysis in OSA patients and OSA severity analysis.

## 1. Introduction

Obstructive sleep apnea (OSA) has been widely reported as a potentially fatal respiratory disease that occurs in sleep. It is characterized by recurrent disorder of upper respiratory tract, with clinical manifestations of daytime sleepiness, snoring, and decreased sleep quality [1]. OSA was reported to have autonomic nerve system (ANS) dysfunction, and can lead to a range of complications, such as respiratory system disease, cerebrovascular disease, and even sudden death [2,3]. Therefore, in practice, ANS evaluation of OSA patients is considered essential [4].

Heart rate variability (HRV) is a kind of noninvasive method which can effectively evaluate ANS function in OSA patients [5,6,7]. The classical HRV analysis methods include time and frequency domain HRV analysis. HRV frequency domain analysis can better evaluate the relationship between HRV and ANS system [8,9]. HRV time/frequency domain analysis method is based on the assumption that signal is linear, while biological processes in human body are nonlinear processes [10]. Nonlinear analysis methods have received extensive attention in recent years. Complexity of human physiological system can be quantitatively analyzed by nonlinear methods, such as Princare graph [11], correlation dimension [12], and complexity analysis [13]. HRV nonlinear analysis can be used to evaluate the disturbance degree of ANS. Especially, the entropy methods are considered as reliable methods to estimate the complexity of ANS [14].

In frequency domain analysis, low frequency power (LF) and high frequency power (HF) index can indicate the balance controlling of ANS. Song et al. found that the LF/HF index displayed the best statistical significance in HRV indices (*p* < 0.05) [15]. Urbanik et al. reported significant differences between groups in the LF/HF values, which were larger in OSA subjects compared with in normal subjects (*p* < 0.05) [16]. Park et al. reported a significant positive correlation between LF/HF and Apnea Hypopnea Index (AHI) (r = 0.610, *p* < 0.001), and LF/HF was regarded as the most useful index to evaluate the AHI degree of OSA patients [17]. It can be observed that the LF/HF performed better in HRV analysis, but LF/HF only reflects the ANS balance as a whole in a certain period of time.

Entropy methods can be used to estimate the complexity of HRV. In general, normal systems have inherent complexity, while the complexity of system in most patients has downward trends [18,19,20]. Haitham et al. applied sample entropy method to study HRV of OSA subjects, and results revealed that HRV pattern of OSA patient was less complex than that of normal subject [21]. Pan et al. reported that multiscale entropy could successfully distinguish normal subjects from OSA patients (*p* < 0.05) [22]. As a method suitable for complexity analysis of time series in biological systems, permutation entropy directly reflects the time information contained in time series. Ravelo et al. found that adding permutation entropy can improve the classifier performance of OSA detection [23]. These findings supported that complexity of the original RR sequence can be used for analysis of HRV pattern changes in OSA patients.

However, the changes of ANS in OSA patients are also affected by daily activities and individual differences [24]. The short-term fluctuation of ANS can reflect the changes caused by OSA and reduce the interference of individual differences and self-changes to a certain extent. Research revealed that most studies focused on the overall complexity evaluation of the HRV, ignoring the short-term dynamic change of HRV. Considering that the fluctuations of respiratory and cardiovascular may lead to HRV disturbance, short-term dynamic changes of ANS in OSA patients may reflect important pathological information [25]. In previous studies, we proposed sliding trend fuzzy apnea entropy based on empirical mode decomposition to reflect the complexity of short-term sympathetic changes [26]. But, as a whole, sympathetic and parasympathetic nerves of the ANS antagonize and coordinate the physiological activities of the human body. The complexity of short-term ANS changes in OSA patients should be further explored.

Therefore, the variance delay fuzzy approximate entropy (VD_fApEn) was proposed in this paper. It is a nonlinear HRV analysis method which can be used for further dynamic analysis of the ANS in OSA patients. First, the small-scale variance of successive RR sequences was firstly extracted to reflect the tension of ANS. Then, by applying fuzzy entropy (fApEn) combined with delay method, VD_fApEn was calculated reflecting the disorders of short-term fluctuations in ANS. Finally, the VD_fApEn was used for OSA screening and analysis of OSA severity. Further details will be given later in this article.

## 2. Methods

### 2.1. Data

The database used in this paper for OSA analysis was downloaded from www.physionet.org, which provides the Apnea-ECG data on obstructive sleep apnea. This database contains 70 single-lead ECG signals with 100 Hz sampling frequency, and the lengths varying between 401 and 587 min. Each recording includes minute-by-minute annotations to indicate the presence or absence of apnea during that minute, which were made by human experts.

According to the number of minutes with apnea, the recordings in the PhysioNet database were classified into three categories: A, B, and C. Recordings having fewer than 5 min of disordered breathing were defined as class C (control group, total 6 males and 5 females, aged from 27–42 years). Recordings containing at least 1 h with an apnea index of 10 or more and at least 100 min of apnea were classified as class A (apnea group, total 15 males and 1 female, aged from 29–63 years). Between these two groups, recordings containing at least 1 h with an apnea index of 5 or more and between 5 and 99 min with apnea during the recording were defined as class B (borderline apnea group, 10 recordings, total 4 males and 1 female, aged from 39–53 years).

According to these criteria, 70 recordings of 32 subjects were classified as follows: 40 class A recordings, 10 class B recordings, and 20 class C recordings. In this study, 10 borderline recordings of class B were excluded, and a total of 60 recordings from class A (20 recordings) and class C (40 recordings) were used. Apnea-hypopnea index (AHI) is an indicator of the severity of OSA, defined as the number of apneas and hypopnea events per hour during sleep. The 60 recordings were further divided as following groups according to AHI as follows: normal group (20 recordings; AHI < 5), mild-moderate OSA group (14 recordings; 5 ≤ AHI < 30), and severe OSA group (26 recordings; AHI ≥ 30).

### 2.2. HRV Analysis Method

**Figure 1:**
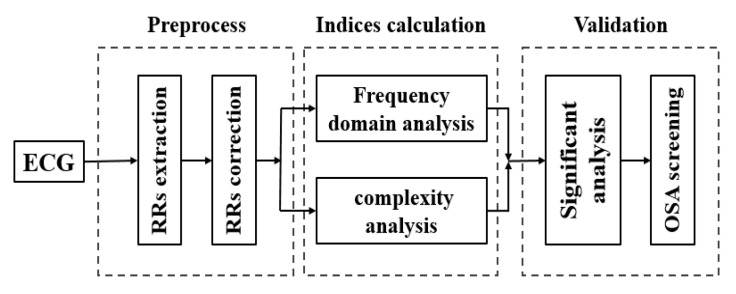
The flow chart of heart rate variability (HRV) analysis in this study.

Figure 1 shows a flow chart of the HRV analysis method in this paper. First, RR intervals were extracted and corrected. Then, frequency domain indices and complexity indices were calculated for short-term HRV measurements. Finally, these indicators were verified by significance analysis.

#### 2.2.1. Preprocess

First, the 60 full-night ECG recordings were intercepted in 6 h for the regularization of data. Then, RR segments (RRs) were extracted from the ECG signals with the algorithm proposed by Pan and Tompkins [27], and the physiologically uninterpretable points were eliminated with the median filter that proposed by Chen et al. [28]. It was reported that 5 min was an appropriate length of time for HRV analysis [26]. The sampling frequency of 5-min RR segments was inverted to 2 Hz via cubic spline interpolation. Each 6-h ECG recording includes 72 RR sequences, with each sequence lasting 5 min. The 5-min HRV indices were calculated, and 72 groups of HRV indices were obtained from each recording. Finally, a set of HRV indices for each recording were obtained by calculating the average value of each HRV indicator of 72 groups.

#### 2.2.2. Frequency Domain Analysis

Low frequency power (LF, 0.04–0.15 Hz), high frequency power (HF, 0.15–0.4 HZ), and the ratio of LF power and HF power (LF/HF) were computed as HRV frequency indices. These indices are obtained with the fast Fourier transform method to calculate the power spectral density of these RRs.

#### 2.2.3. Complexity Analysis

Fuzzy Approximate Entropy (fApEn): The fApEn is an effective and robust algorithm for measuring the complexity of a discrete sequence, used for assessing the complexity of physiological signals [5]. The detailed calculation steps of fuzzy approximate entropy are as follows:

(a) Given an N samples discrete series {s(l),l=1,2,⋯,N}, then construct m dimensional vector sequences $\left\{X_{l}^{m},l=1,2,\cdots ,N-m+1\right\}$ as follows:

(1) $$X_{l}^{m}=\left\{s\left(l\right),s\left(l+1\right),\cdots ,s\left(l+m-1\right)\right\}-s_{0}\left(l\right),l=1,2,\cdots N-m+1.$$

s(l) represents mean value of the m consecutive values.

(2) $$s_{0}\left(l\right)=\frac{1}{m}\sum_{i=0}^{m-1}s\left(l+i\right).$$

(b) The maximum absolute distance $d_{ij}^{m}$ between $X_{i}^{m}$ and $X_{j}^{m}$ is calculated according to:

(3) $$\begin{array}{cc} d_{ij}^{m}=d\left[X_{i}^{m},X_{j}^{m}\right] & =\underset{p\epsilon \left(0,m-1\right)}{\max}\left\{\left|\left(s\left(i+p\right)-s_{0}\left(i\right)\right)-\left(s\left(j+p\right)-s_{0}\left(j\right)\right)\right|\right\}\;i,j\\ \; & =1,2,\cdots ,N-m;i\neq j \end{array}$$

(c) Define the similarity degree $D_{ij}^{m}\left(n,r\right)$ between $X_{i}^{m}$ and $X_{j}^{m}$ through an exponential fuzzy function $\varphi \left(d_{ij}^{m},n,r\right)$. r is width of the border; n determine gradient of the boundary.

(4) $$D_{ij}^{m}\left(n,r\right)=\varphi \left(d_{ij}^{m},n,r\right)=exp\left(-{\left(d_{ij}^{m}\right)}^{n}/r\right)$$

(d) Then, calculate the average similarity from each vector to another as follows:

(5) $$\emptyset^{m}\left(n,r\right)=\frac{1}{N-m}\sum_{i=1}^{N-m}\left(\frac{1}{N-m-1}\sum_{j=1,j\neq i}^{N-m}D_{ij}^{m}\right).$$

(e) Construct m+1 dimensional vector sequences $\left\{X_{k}^{m+1},k=1,2,\cdots ,N-m\right\}$ and repeat (3)–(5), $\emptyset^{m+1}\left(n,r\right)$ as follows:

(6) $$\emptyset^{m+1}\left(n,r\right)=\frac{1}{N-m}\sum_{i=1}^{N-m}\left(\frac{1}{N-m-1}\sum_{j=1,j\neq i}^{N-m}D_{ij}^{m+1}\right).$$

(f) Finally, the fApEn is defined as:

(7) $$FuzzyEn\left(m,n,r\right)=\underset{N\to \infty }{lim}\left[ln\emptyset^{m}\left(n,r\right)-ln\emptyset^{m+1}\left(n,r\right)\right]$$

For a finite discrete series, fApEn can be calculated as:

(8) $$fApEn\left(m,n,r\right)=ln\emptyset^{m}\left(n,r\right)-ln\emptyset^{m+1}\left(n,r\right)$$

**Figure 2:**
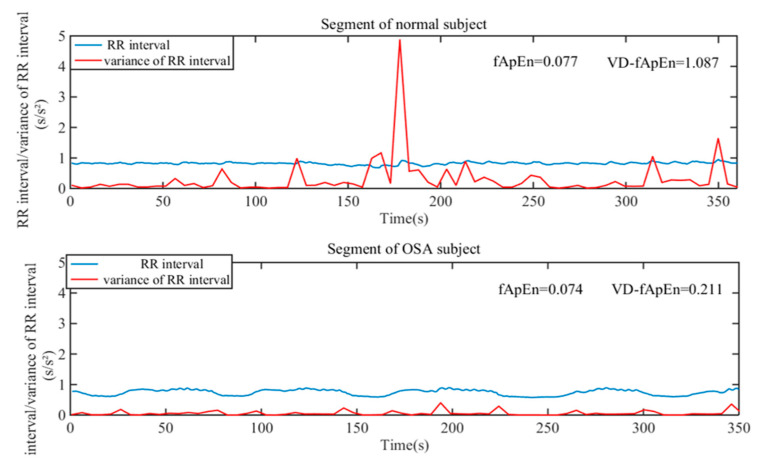
The typical 5-min RR intervals for a segment of normal subject and a segment of obstructive sleep apnea (OSA) subject. The red lines are obtained by calculating the variance of the signal at five points.

Varience Delay Fuzzy Approximate Entropy (VD_fApEn): First, the variance of RR sequence in small scale was calculated to reflect the fluctuation change of HRV. As shown in Figure 2, there was a significant difference in the complexity of RR sequence variance (red line) between OSA patient and normal subject. Thus, we attempted to quantitatively evaluate the complexity of RR sequence variance. Secondly, delay method can weaken the strong correlation between continuous heartbeats, and fApEn method was an improved evaluation of signal complexity [5,29]. Therefore, we applied the fApEn method combined with delay method to calculate the complexity of RR sequence variance. The detailed calculation are as follows:

(a) Given an N samples discrete series V{v(l),l=1,2,⋯,N}, zero-mean normalization of the sequence was performed to obtain a new sequence W{w(l),l=1,2,⋯,N} as follows:

(9) $$w\left(l\right)=\frac{\left(v\left(l\right)-v_{0}\right)}{v_{\sigma }}\;,\;1\leq l\leq N$$

where $v_{0}$ and $v_{\sigma }$ is the mean value and the standard deviation of the series V, respectively.

(b) Coarse-grain W: The original RR series are divided into a series of non-overlapping segments. τ was defined as scale of RR interval, and the variance of each segments was calculated to get a new sequence $\left\{s\left(l\right),l=1,2,\cdots ,\lfloor \frac{N}{\tau }\rfloor \right\}$.

(10) $$s\left(l\right)=\frac{1}{\tau }\sum_{i=\left(l-1\right)\tau +1}^{l\tau }{\left(w\left(i\right)-w_{l}\right)}^{2}\;,\;1\leq l\leq \lfloor \frac{N}{\tau }\rfloor =C,$$

(11) $$w_{l}=\frac{1}{\tau }\sum_{i=\left(l-1\right)\tau +1}^{l\tau }w\left(i\right).$$

(c) To embed the delay method into the fApEn method, we constructed m dimensional vector sequences $\left\{X_{l}^{m}\left(\delta \right),l=1,2,\cdots ,N-m+1\right\}$ with a time delay δ as:

(12) $$X_{l}^{m}\left(\delta \right)=\left\{s\left(l\right),s\left(l+\delta \right),\cdots ,s\left(l+\left(m-1\right)\delta \right)\right\},l=1,2,\cdots ,C-\left(m-1\right)\delta$$

The following steps are the same as the fApEn calculation steps (2)–(7).

### 2.3. Simulation Test

Considering that the ANS is highly nonlinear and complex, a nonlinear signal system model was constructed to verify the feasibility of VD_fApEn for ANS analysis. In addition, it is necessary to choose appropriate *N* and *r* for entropy indices of the HRV signal. Therefore, the performances of fApEn and VD_fApEn with changes of parameters were compared using simulated signals in this study. Three kinds of nonlinear signals with different complexity are generated as *MIX* (0.1, 0.5, 0.9), which are defined as follows:

(13) $$MIX{\left(p\right)}_{i}=\left(1-Z_{i}\right)X_{i}+Z_{i}Y_{i}$$

(14) $$X_{i}=2sin\left(2\pi i∕12\right)$$

where $Y_{i}$ is a random variable uniformly distributed between −$\sqrt{3}$ and $\sqrt{3}$, $Z_{i}$ is random variables, and the probability of $Z_{i}$ = 1 is p, while the probability of $Z_{i}=0$ is 1−p. i is the number of $MIX{\left(p\right)}_{i}$, which was set as 1000. By increasing the value of p, the randomness and complexity of MIX(p) are increased, making the complexity of generating signals increase.

In the calculation of entropy, three parameters, *m*, *N*, and *r*, should be focused. *m* is the dimension of vector sequences and was proved to be suitable set as 2 [26,30]. To find the most appropriate *N* and *r* settings in the complexity measure, we compared the variation of fApEn and VD_fApEn with *N* and *r*. For the selection of parameter *N*, MIX(p) was divided into sequences of 100 to 1000 points, and the r was fixed into value 0.2. For the selection of parameter *r*, MIX(p) was fixed into 500 points, and *r* ranged from 0.1 to 1.

### 2.4. Indices Validation

HRV indices were calculated using MATLAB (R2014b, Mathworks, Natick, MA, USA). All statistical analyses were adopted using SPSS (version 22.0.0.0 SPSS, Inc., Chicago, IL, USA). The HRV indices data calculated in this study were analyzed in SPSS, and results showed that the data conforms to normal distribution. HRV indices were evaluated by one-way ANOVA followed by post hoc analysis with the least significant difference (LSD) test for analysis among the control group, mild-moderate OSA group, and severe OSA group. *p* < 0.05 represents statistically significant, and the statistical results of indices are showed as mean ± SD. For OSA screening, Fisher’s discriminant function of SPSS was used, and the overall accuracy, sensitivity, and specificity of HRV indices were presented [31].

## 3. Results

### 3.1. Simulation Results Comparisons of Indices

Monotonicity, consistency, and continuity are important references used in complexity analysis to reflect the performance of entropy indices. Monotonicity refers to the monotonic tendency of parameter variation; consistency means that if a sequence is more complex, all values of entropy for testing should be larger; continuity means that there should be no mutation when the parameters change slightly [32].

**Figure 3:**
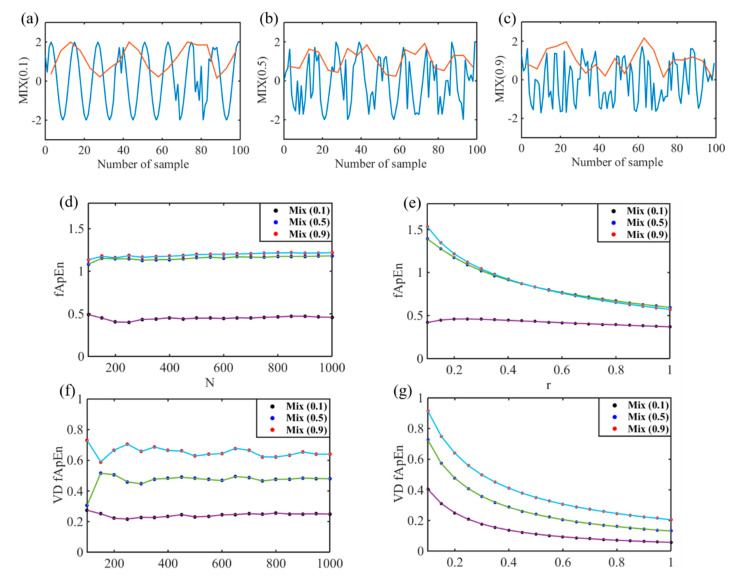
One hundred-point segments are randomly selected from 1000 simulated (**a**) MIX (0.1), (**b**) MIX (0.5), and (**c**) MIX (0.9) signals; red lines are obtained by calculating the variance of the signal at 5 points; when N increases from 200 to 1000 at each increase of 50 (r = 0.20), there is a change of (**d**) fuzzy approximate entropy (fApEn) and (**f**) variance delay (VD)_fApEn; when r increases from 0.1 to 1 at each increase of 0.05, there is a change of (**e**) fApEn and (**g**) VD_fApEn.

Figure 3 presents the results of parameters selection and comparison of ApEn and VD_fApEn. From Figure 3a–c, as complexity of the signal (the blue line) increased, complexity of the signal’s short-term variance (the red line) also increased gradually. As shown in Figure 3d–f, when the *n* value is greater than 200, both fApEn and VD_fApEn reached relatively stable. VD_fApEn made a better distinction among Mix (0.1, 0.5, 0.9) signals. As shown in Figure 3e,g, there was a more pronounced monotonic downward trend with increasing r in VD_fApEn. Moreover, there were no crossover of VD_fApEn, whereas crossovers were found in fApEn. Among simulated signals, index VD_fApEn showed better monotonicity, consistency and continuity.

### 3.2. Disease Severity Analisis of OSA

**Figure 4:**
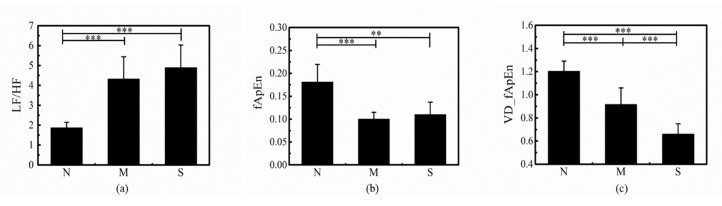
Comparison of the (**a**) low frequency (LF)/high frequency (HF) ratio, (**b**) nonlinear index fApEn, (**c**) VD_fApEn for the normal, mild/moderate OSA, and severe OSA groups.

The results of frequency domain and nonlinear indices for the normal, mild-moderate OSA and severe OSA group are shown in Table 1. There were no significant differences in LF and HF among these three groups. As shown in Figure 4a,b, LF/HF and fApEn could significantly distinguish between normal group and mild-moderate OSA group, and between normal group and severe OSA group. In Figure 4a, LF/HF values showed an increased trend from normal to mild-moderate OSA to severe OSA group. Figure 4c shows a down trend VD_fApEn values from normal to mild-moderate OSA to severe OSA group. In addition, the differences of VD_fApEn in any two of the three groups were statistically significant. Therefore, VD_fApEn can be used for OSA disease severity analysis.

### 3.3. OSA Screening

As shown in Table 2, the OSA screening results were also calculated for each individual using SPSS. LF/HF (80.0% and 70.0%) had a higher accuracy and sensitivity compared with fApEn (78.3% and 82.5%), while VD_fApEn reached the highest accuracy and sensitivity for 90.0% and 87.5%.

**Figure 5:**
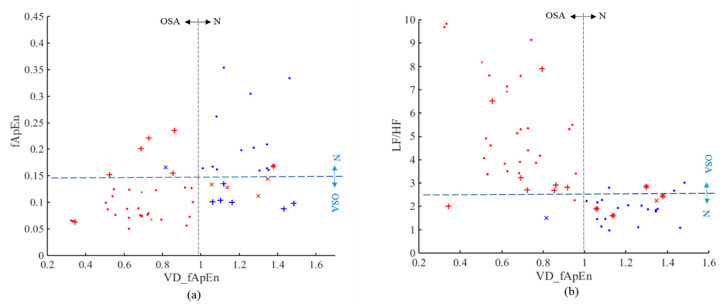
(**a**) The comparison of VD_fApEn and fApEn for each OSA patient screening; blue/red dot: normal/OSA recording correctly detected; red/blue+: the fApEn has misclassified the OSA/normal recording; red/blue; ×x: the VD_fApEn has misclassified the OSA/normal recording; red *: both indicators misclassify the OSA recording; (**b**) The comparison of VD_fApEn and LF/HF ratio for each OSA patient screening; blue/red dot: normal/OSA recording correctly detected; red/blue+: the LF/HF has misclassified the OSA/normal recording; red/blue ×: the VD_fApEn misclassified the OSA/normal recording; red *: both indicators misclassify the OSA recording; N:normal subject; OSA: OSA patient.

Figure 5 shows the result of each OSA recording screening with these three indices. In Figure 5a, there were 12 misclassifications (6 normal, 6 OSA) for fApEn, 5 misclassifications (1 normal, 4 OSA) for the VD_fApEn, and 1 misclassification (1 OSA) of both indicators. In Figure 5b, there were 8 misclassifications (OSA) for LF/HF, 2 misclassifications (1 normal, 1 OSA) for the VD_fApEn, and 4 misclassifications (4 OSA) of both indicators. Moreover, Figure 5a,b show that the red and blue symbols in the left and right direction are easier to separate, indicating the screening ability of VD_fApEn is better.

### 3.4. Analysis of Different Scales for RR Intervals

In calculation of the VD_fApEn, variance of RR sequence in small scale was calculated to reflect the fluctuation change of HRV. In this study, the results of VD_fApEn applied to OSA disease severities analysis, and the results of OSA disease screening were analyzed, as RR scale ranged from 2 to 9.

**Figure 6:**
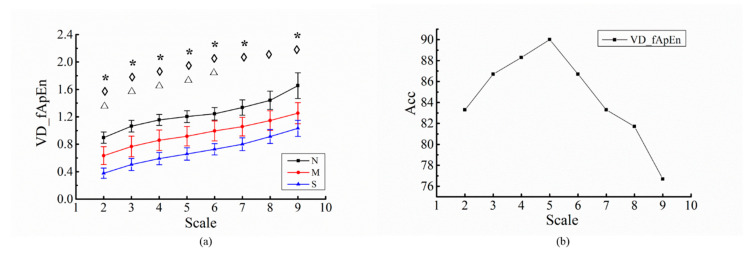
(**a**) Relationship between VD_fApEn and different RRs scales for three groups; (**b**) analysis of relationship between OSA screening accuracy and different scales. The black line is the control group; red line is the mild-moderate OSA group; the blue line is the severe OSA group. *: significant difference exist between N and M (*p* < 0.05); ◊: significant difference exist between N and S (*p* < 0.05); Δ: significant difference exist between M and S (*p* < 0.05).

Figure 6 shows the result of relationship between performance of VD_fApEn and different RR intervals scales. In Figure 6a, the values of VD_fApEn for normal, mild-moderate OSA and severe OSA group had an upward trend with increase of scales. VD_fApEn can significantly distinguish any two of the three groups when scales of RR intervals was limited as 2 to 6.

In Figure 6b, when the sequence scale rose from scale 2 to 5, the OSA screening accuracy of the VD_fApEn reached its maximum and decreased when the sequence scale rose from 5 to 9. In conclusion, VD_fApEn analysis of OSA is considered to be effective, and OSA patients with different severities could be distinguished when RR interval was 5.

## 4. Discussion

### 4.1. Comparison and Summary

This paper verified the performance of HRV frequency domain indices by comparing with previous studies. LF/HF can reflect the status of autonomic nerve control and is regarded as a reliable indicator providing valuable information in apnea events discrimination [32]. Results revealed that the LF/HF could significantly distinguished between the normal group and OSA group (mild-moderate and severe OSA group) (Table 1, Figure 4). We compared our work with previous studies and validated the performance of the LF/HF. Results in this study are consistent with the findings of previous relative studies [15,17,26], and we selected LF/HF for comparison with nonlinear HRV indices. However, as in most studies, LF/HF was unable to distinguish among different severities of OSA [7,22,33].

In this study, VD_fApEn could significantly differentiate between any two groups among normal, mild-moderate OSA and severe OSA group compared with LF/HF and fApEn (Table 1, Figure 4). We also analyzed the correlation between the HRV indices (VD_fApEn and LF/HF) and the AHI. Results showed that the index VD_fApEn and AHI were significantly negative correlated (r = −0.7430, *p* < 0.001), while the index LF/HF and AHI were positively correlated (r = 0.6504, *p* < 0.001). It can be observed that there is a better correlation between VD_fApEn and AHI. In addition, HRV indices have been used for OSA screening in previous studies. Haitham et al. reported that sample entropy of OSA patients had less complex HRV pattern, and the accuracy of sample entropy for OSA screening reached 70.3% [21]. The sliding trend fuzzy approximate entropy (SlTr-fApEn), which is based on the empirical mode decomposition method, was proposed in our previous study to reflect the complexity of short-term sympathetic changes. The result for OSA screening showed an improved accuracy (85.0%) [26]. In this study, VD_fApEn had a higher accuracy (90.0%) compared with the LF/HF (80.0%) or compared with fApEn (78.3%) (Table 2). Briefly, VD_fApEn was seen as an effective indicator for OSA screening and disease severity analysis.

### 4.2. Method Proposed and Parameter Selection

HRV analysis is widely adopted in ANS function evaluation, but the instantaneous change of ANS was ignored. Considering that HRV is the result of many nonlinearly interacting processes, nonlinear HRV methods are suitable for HRV analysis. As shown in Figure 2, two typical examples of 5-min RR sequences in normal subject and OSA patient were displayed. Results showed that the complexity difference of small-scale variance for these two RR sequences is significant. In addition, three nonlinear signals with different complexities were simulated in Figure 3, and results showed that the short-term variance complexity analysis was more suitable for nonlinear and nonstationary system. Therefore, we attempted to calculate the complexity of short-term RR sequence variance to analyze the instantaneous change of ANS in OSA patients.

In this study, the small-scale variances of successive RR sequences were extracted to reflect the instantaneous tension of ANS, and fApEn combined with delay method was adopted to reflect the complexity of ANS tension. Studies have shown that the strong correlation between continuous heartbeat may mask the non-linear measurement, and the occurrence of apnea events may have a delayed effect on ANS [29]. Thus, delay algorithm may better evaluate the effect of apnea events on ANS. Moreover, as a nonlinear method, entropy method has been widely concerned for the complexity measurement of HRV [6,30,34]. The fApEn method has been proven to improve the evaluation of signal complexity [5,35]. Therefore, delay algorithm was embedded into fApEn method for complexity calculation of ANS tension.

Parameters of VD_fApEn were also analyzed in this study. The scale of RR sequences for calculating variance was analyzed, and it could be inferred from the results that the ability of VD_fApEn to distinguish OSA disease severity increased with scale decreased. When the scale reduced to 5, the OSA screening accuracy of VD_fApEn reached its maximum (90%) (Figure 6). Therefore, 5 consecutive heartbeats may be suitable for instantaneous change assessment of ANS, and 5 points was adopted as the scale of variance in VD_fApEn. In addition, the parameters of entropy m and r were set as 2 and 0.25, respectively, which were consistent with other findings [30,36].

### 4.3. Physiological Significance

OSA is a common respiratory cardiovascular syndrome during sleep. It has been extensively studied that abnormality of ANS existed in OSA patients, and HRV analysis was reported to be an effective method to evaluate the function of ANS [37]. Considering that maintaining normal HRV is associated with autonomic regulation of the ANS, reduced HRV in OSA patients may reflect disorders of ANS function. The classical HRV index LF/HF is related to the overall balance of autonomic nerves. In this study, increased LF/HF value can be observed in OSA patients (Table 1, Figure 4). This result indicated that the ANS imbalance was significantly increased in OSA patients, which is caused by increased sympathetic activity.

Research showed that the fluctuation of respiratory and cardiovascular may lead to disorder of HRV, and OSA was related to the activity and short-term state changes of the ANS [38]. It can be inferred that the short-term changes in ANS of OSA patients may reflect more physiological information. Because the variance in small scale can reflect fluctuation change of short-term series, and fApEn embedded with delay method can evaluate the complexity of sequences. VD-fApEn can reveal the complexity of fluctuations within the ANS, and it also provides a new perspective for ANS analysis of OSA patients. Results indicate that VD-fApEn value decreased with the increase of OSA disease severity, reflecting that the complexity of fluctuations in ANS decreased significantly when the severity of OSA increased (Table 1, Figure 4). This may due to decreased tension and activity of the ANS in OSA patients, resulting in decreased ability of the ANS to adapt to external changes.

Present study still has some limitations for further improvement. First, personal factors of subjects may have impact on the results (such as age and body mass index, etc.). Second, our algorithm should be improved in many ways, the proposed index also needs further verification. Finally, the existence of underlying cardiovascular disease and sleep related factors may have a certain impact on the HRV analysis of OSA patients, which was not considered. In future study, we will adopt a larger amount of data and further verify the reliability of the index proposed in this paper by combining polysomnography (PSG) indicators.

## 5. Conclusions

In this study, variance delay fuzzy approximate entropy (VD_fApEn) was proposed as a nonlinear index to study the fluctuation change of ANS in OSA patients. When used for disease severity analysis of OSA, results showed that VD_fApEn could significantly differentiate between any two groups among the normal, mild-moderate OSA, and severe OSA group. The complexity of autonomic fluctuations decreased significantly with increased severity of OSA. When used for OSA screening, VD_fApEn had a higher accuracy compared with the traditional HRV indices. Therefore, VD_fApEn provides a potential clinical method for analysis of ANS alterations in OSA patients and severity of OSA disease.

## Figures and Tables

**Table 1:** Frequency domain indices and nonlinear indices of HRV for disease severity analysis.

Indices		N	M	S	*p*		
					N&M	N&S	M&S
Frequency domain indices	LF	0.004 ± 0.005	0.001 ± 0.0004	0.011 ± 0.025	0.638	0.190	0.097
	HF	0.004 ± 0.005	0.0004 ± 0.0003	0.009 ± 0.028	0.642	0.315	0.166
	LF/HF	1.862 ± 0.576	4.325 ± 2.238	4.893 ± 2.279	0 ***	0 ***	0.367
Nonlinear indices	fApEn	0.181 ± 0.077	0.100 ± 0.03	0.11 ± 0.054	0 ***	0.001**	0.407
	VD_fApEn	1.204 ± 0.172	0.917 ± 0.283	0.659 ± 0.179	0 ***	0 ***	0 ***

N: normal group; M: mild-moderate OSA group; S: severe OSA group; *, **, *** represent *p* < 0.05, *p* < 0.01, and *p* < 0.001, respectively.

**Table 2:** Performance of indices for OSA screening.

	TP	TN	FP	FN	Accuracy	Sensitivity	Specificity
LF/HF	28	20	0	12	80	70	100
fApEn	33	14	6	7	78.3	82.5	70
VD_fApEn	35	19	1	5	90	87.5	95

TP: true positive; TN: true negative; FP: false positive; FN: false negative.
